# Directed Therapy: An Approach to the Improved Treatment of Exfoliation syndrome

**DOI:** 10.4103/0974-9233.48866

**Published:** 2009

**Authors:** Allison Angelilli, Robert Ritch

**Affiliations:** 1 From Einhorn Clinical Research Center, New York Eye and Ear Infirmary, United States of America; 2 From Department of Ophthalmology, New York Medical College, Valhalla, New York, NY, United States of America

**Keywords:** Exfoliation Syndrome, Pseudoexfoliation, Glaucoma, Directed Therapy, Miotics, Aqueous Suppressants

## Abstract

Exfoliation syndrome (XFS) is an age-related, generalized disorder of the extracellular matrix characterized by the production and progressive accumulation of a fibrillar extracellular material in many ocular tissues and is the most common identifiable cause of open-angle glaucoma worldwide. Exfoliation syndrome plays an etiologic role in open-angle glaucoma, angle-closure glaucoma, cataract, and retinal vein occlusion. It is accompanied by an increase in serious complications at the time of cataract extraction, such as zonular dialysis, capsular rupture, and vitreous loss. It is associated systemically with an increasing number of vascular disorders, hearing loss, and Alzheimer's disease. Exfoliation syndrome appears to be a disease of elastic tissue microfibrils. Directed therapy simply means devising specific treatments for specific diseases. There was little incentive to attempt to distinguish between various open-angle glaucomas if the treatments were essentially the same. However, this view also prevented the application of directed therapy in those instances in which such was available and applicable. Pilocarpine has multiple beneficial actions in eyes with XFS. Not only does it lower IOP, but by increasing aqueous outflow, it should enable the trabecular meshwork to clear more rapidly, and by limiting pupillary movement, should slow the progression of the disease. Theoretically, miotics should be the first line of treatment. Pilocarpine 2% q.h.s. can provide sufficient limitation of pupillary mobility without causing these side effects. In 2007, two common single nucleotide polymorphisms in the coding region of the *lysyl oxidase-like 1* (*LOXL1*) gene located on chromosome 15 were specifically associated with XFS and XFG. LOXL1 is a member of the lysyl oxidase family of enzymes, which are essential for the formation, stabilization, maintenance, and remodelling of elastic fibers and prevent age-related loss of elasticity of tissues. LOXL1 protein is a major component of exfoliation deposits and appears to play a role in its accumulation and in concomitant elastotic processes in intra- and extraocular tissues of XFS patients. This discovery should open the way to new approaches and directions of therapy for this protean disorder.

Exfoliation syndrome (XFS) is an age-related disease of the extracellular matrix characterized by the production and deposition of a fibrillar extracellular material in numerous ocular tissues. It is now recognized as the most common identifiable cause of open-angle glaucoma, affecting an estimated 60 to 70 million people worldwide.^1^,^2^ The reported prevalence of XFS both with and without glaucoma has varied widely. This reflects true differences due to racial, ethnic, or other as-yet-unknown factors; the age and sex distribution of the patient cohort or population group examined; the clinical criteria used to diagnose XFS; the ability of the examiner to detect early stages and/or more subtle signs; the method and thoroughness of the examination; and the awareness of the observer.

Exfoliation syndrome is diagnosed by the presence of typical exfoliation material (XFM) on the anterior lens surface or pupillary border. All anterior segment structures are involved. The classic pattern of deposition on the lens consists of three distinct zones that become visible when the pupil is fully dilated, a central disc, intermediate clear zone created by the iris rubbing XFM from the lens surface during its physiologic excursions, and a granular peripheral zone. The material on the anterior lens capsule causes rupture of iris pigment epithelial cells leading to pigment dispersion, trabecular meshwork hyperpigmentation, iris transillumination defects at the pupillary margin and loss of the pupillary ruff. Pigment dispersion after dilation can lead to marked IOP rise and may be one of the earliest manifestations of the disease. Exfoliation material is often found at the pupillary border.

Patients with XFS are twice as likely to convert from ocular hypertension to detectable glaucoma.^3^ Exfoliative glaucoma (XFG) is more severe than primary open angle glaucoma (POAG). There is greater diurnal intraocular pressure (IOP) fluctuation, greater visual field loss and optic nerve head damage at presentation, poorer response to medications, faster visual field progression and increased need for surgical intervention. At any given IOP, eyes with XFS are at significantly greater risk for glaucomatous damage, suggesting that pressure-independent factors play a significant role in visual field loss in these patients.

The prevalence of XFS increases with age in all affected populations. Approximately two-thirds of patients have unilateral disease on clinical examination, however, XFS is often detectable in the contralateral eye with conjunctival biopsy.^4^ This finding supports the hypothesis that the development of XFS is similar to uveitis in that an underlying predisposition plus an environmental or immunologic trigger leads to disease manifestation.

## PATHOGENESIS OF XFS

The pathogenesis of XFS is defined by the accumulation of an abnormal fibrillar material biochemically related to the composition of the extracellular matrix that results either from excessive production and/or insufficient breakdown. The product has a unique structure as seen by electron microscopy.^5^ Streeten et al first hypothesized that XFS is an elastosis based on similarities of XFM and zonular fibers.^6^ These characteristic fibrils, composed of microfibrillar subunits surrounded by an amorphous matrix, contain epitopes of elastic fibers such as fibrillin-1, elastin, tropoelastin, amyloid P and microfibril-associated glycoprotein, vitronectin and the cross-linking enzyme lysyl-oxidase.^7^,^8^

Two common single nucleotide polymorphisms (SNPs) in the coding region of the lysyl-oxidase-like 1 (LOXL1) gene on chromosome 15 have been identified and account for virtually all cases of XFS in several populations.^9^–^12^ While the SNPs confer susceptibility to XFS, not all people with the SNPs will develop the disease.

LOXL1 is a member of the lysyl oxidase enzyme family responsible for formation, stabilization and remodeling of elastic fibers. They are important for cross-linking of elastin, for providing a scaffold that ensures spatially defined deposition of elastin and for regulating the production of elastin.^13^,^14^ The currently proposed pathogenesis of XFS is a stress-related, excessive production by elastogenic cells of elastic microfibrils that aggregate into a typical configuration via an enzymatic cross-linking process.^15^ There is evidence that derangement of the balance of matrix metalloproteinases and their tissue inhibitors, increased oxidative stress and an impaired stress response contribute to impaired degradation and accumulation of fibers.^16^–^19^ These fibers impair trabecular meshwork outflow resulting in an increased rise of IOP.

## OCULAR AND SYSTEMIC ASSOCIATIONS

XFS is associated with both chronic open-angle glaucoma and angle closure. The zonules and ciliary body are often diffusely coated with XFM and zonules are often frayed and broken. Zonular fragility and poor pupillary dilation contribute to an increased incidence of serious complications during cataract extraction, including zonular dehiscence and vitreous loss. Phacodonesis and even subluxation of the lens or intraocular lens is common.

It appears that XFS is etiologically related to cataract formation itself. Decreased aqueous humor ascorbic acid concentrations in XFS,^20^ increased malondialdehyde concentrations^21^ and increased 8-iso-prostaglandin F_2a_^22^ suggest that free radicals and oxidative damage play a role in the disease process. There is also breakdown of the blood-aqueous barrier with resultant increased protein concentrations in the anterior chamber.

Ocular ischemia is clearly correlated with XFS. An increased frequency of retinal vein occlusion has been noted in XFS^23^–^25^ and iris hypoperfusion can be detected angiographically and histopathologically.^26^,^27^ Patchy iris neovascularization can be seen when capillary dropout is extensive.^7^,^15^ Deposits of XFM can be found in the walls of vortex veins and central retinal veins. Not surprisingly, cardiovascular and cerebrovascular disorders are emerging as systemic associations of XFS. XFM has been found electron microscopically in the heart, liver, lungs, kidney and meninges in patients with XFS.^28^,^29^ Ocular, retrobulbar, cerebral and carotid blood flow are reduced.^30^–^34^ XFS is a risk factor for cardiovascular disease^35^ and several reports have linked XFS with Alzheimer's disease, hearing loss and transient ischemic attacks.^36^–^39^ The pathologic connection between XFM and the functional deficits seen is currently unclear.

### II. Directed Therapy for XFS

As the term implies, directed therapy aims to specifically address and interfere with the underlying disease pathogenesis. Ophthalmologists have begun to recognize the inadequacy of therapy limited solely to IOP reduction, particularly with the significant potential morbidities associated with XFS. The widespread systemic and ocular manifestations of XFS underscore the need for a broad and comprehensive treatment paradigm. It is essential that future investigations focus on disease-specific therapeutic modalities.

## DIRECTED OCULAR ANTIHYPERTENSIVE THERAPY

The management of XFS currently hinges on ocular antihypertensive therapy and initially can be achieved with topical therapy in many cases. Prostaglandin analogues such as latanoprost and travoprost reduce IOP at each time point in the 24-hour diurnal curve as compared to untreated IOP, with travoprost conferring a slightly higher level of reduction.^40^ Latanoprost reduced IOP and narrowed the range of diurnal fluctuation in comparison to timolol in XFS.^41^

Pilocarpine has multiple beneficial actions in eyes with XFS. Not only does it lower IOP, but by increasing aqueous outflow, it should enable the trabecular meshwork to clear more rapidly, and by limiting pupillary movement, should slow disease progression. Theoretically, miotics should be the first line of treatment. Prostaglandin analogues are complementary in increasing uveoscleral outflow facility. By decreasing outflow, aqueous suppressants may cause longterm worsening of trabecular function that may be particularly detrimental in XFS.^42^

Unfortunately, pilocarpine is underutilized in the treatment of glaucoma due to the possibility of decreased vision or the misconception that it must be used q.i.d. It is our experience that pilocarpine 2% q.h.s. can provide significant limitation of pupillary movement without the inconvenience of q.i.d. dosing and dimming of vision. Pilocarpine can also blunt an early morning IOP spike.^43^

Argon laser trabeculoplasty is particularly effective in XFS.^44^ Primary ALT can delay the need for topical therapy in some patients, although there is a general decrease in efficacy over time, with 35-55% remaining medication free at 3-5 years.^45^ A minority of patients will experience a sudden and dramatic rise in IOP within two years following treatment, presumably due to continued pigment liberation overwhelming the meshwork. Pilocarpine 2% q.h.s. may provide protection from this. Limited data on selective laser trabeculoplasty (SLT) suggests that success may be comparable, although prospective studies are necessary to confirm this and also to compare results with argon laser trabeculoplasty.^46^ Marked IOP elevations associated with pigment release in patients with heavily pigmented trabecular meshworks necessitating filtration surgery may occur after SLT.^47^ Trabeculectomy with antifibrotic therapy leads to comparable IOP reduction in XFS and OAG, and offers greater 24-hour IOP control than medical therapy alone.^48^

The investigation of surgical therapy directed at trabecular dysfunction is particularly sensible for XFS. Trabeculotomy has been reported as successful in XFS although additional trials are required.^49^–^51^ Goniotomy is also successful (Kim JH, Sbeity Z, Ritch R: Long-term results of goniotomy and cataract surgery for exfoliative glaucoma. Southeast Asian Glaucoma Interest Group, Seoul, Republic of Korea, Sept. 25, 2008.) Jacobi and Krieglstein^52^ described a procedure in which trabecular aspiration improves outflow facility.

## NOVEL THERAPIES TARGETING TRABECULAR MESHWORK

Drugs that alter the structure of the trabecular meshwork may be particularly effective in XFS. Latrunculin B is currently being evaluated in human trials for its ability to bind to free actin in trabecular cells leading, to temporary disintegration of the existing actin cytoskeletin thereby increasing aqueous outflow.^53^,^54^ Unlike previous compounds with similar mechanisms of action such as sodium-EDTA and cytochalasin B,^55^,^56^ Latrunculin has no known adverse effects on retinal and corneal function.^57^–^59^ Ideally, latrunculin would be effective as a depot medication with once a week or once a month dosing.

## TREATMENT DIRECTED AT XFM

Future therapy of XFS should be directed at preventing formation of XFM or depolymerizing existing XFM. An intriguing line of research is an investigation of the factors that protect the contralateral eye from developing XFS. If the development of the disease in the second eye is actively suppressed, as by an immune mechanism, then elucidation of this could lead to a novel treatment. The systemic ramifications of XFS and the morbidity associated with these diseases highlight the need for a therapy that prevents aggregation of XFM microfibrils disaggregating the microfibrils.

Homocysteine is an amino acid, elevations of which result from a disturbance of methionine metabolism. Hyperhomocysteinemia is a widely recognized cardiovascular risk factor and is reversible with dietary supplementation with folic acid, pyridoxine and cyanocobalamine.^60^,^61^ Hyperhomocysteinemia is associated with disruption of the elastic fibers of the extracellular matrix leading to vascular disease.^62^ Elevated homocysteine levels are present in blood, aqueous and tears of patients with XFS, and there are decreased serum levels of cyanocobalamin, pyridoxine and folic acid.^63^–^66^ The concept of a relationship between hyperhomocysteinemia is supported by the propensity for cardiovascular and cerebrovascular disease in patients with XFS. By the same virtue, it is possible treatment of hyperhomocysteinemia could be beneficial in XFS as it is in cardiovascular disease.

The discovery of the role of LOXL1 polymorphisms in XFS draws attention towards lysyl oxidase and its role in XFM production. Lysyl oxidase is inhibited by homocysteine in vascular endothelia and is also regulated by transforming growth factor-fl 1 (TGF-fl 1).^67^,^14^ Levels of TGF-fl 1 are significantly increased in the aqueous humor of XFS and TGF- fl1 may be responsible for overproduction of extracellular matrix in XFS.^68^ Further information on the biochemical pathway of the production of XFM is critical if we hope to modify the treatment of XFS significantly.

## TREATMENT TARGETING NON-PRESSURE DEPENDENT MECHANISMS

Patients with XFS have an increased likelihood to have coexisting cerebrovascular and cardiovascular disease, and systemic agents that provide neuroprotection and vasoprotection may prove to be an important therapeutic tactic in the treatment of XFS. Pharmacologic treatment of non-IOP dependent mechanisms in glaucoma has traditionally been limited to calcium channel blockers that are widely used in treatment of hypertension, coronary artery disease, migraines and Raynaud's disease. The effect of calcium channel blockers on visual field progression and ocular blood flow is disputed.^69^–^71^

Gingko biloba extract (GBE) has been used in Chinese medicine since 3000 BC for the treatment of aging, dementia, tinnitus, bronchoconstriction and sexual dysfunction with minimal side effects. It improves peripheral, cerebral and ocular blood flow, prevents platelet aggregation and protects against free radical damage.^72^,^73^ Neurons in tissue culture are protected from toxicity-induced apoptosis with GBE, and there is mixed evidence that GBE improves function in patients with Alzheimer's disease and vascular dementia.^74^,^75^ Further elucidation by prospective trials of GBE in glaucoma is warranted.

Curcumin is an anti-oxidant extracted from the plant *Curcuma longa* and is a component of the commonly used Indian spice turmeric. Curcumin has antioxidant, anti-inflammatory, anti-angiogenic and anti-neoplastic activity via the inhibition of numerous mediators of inflammation.^76^–^78^ Clinical trials for the treatment of gastrointestinal and other neoplasms as well as Alzheimer's disease are under way. Common therapeutic doses cause only mild gastrointestinal discomfort in a minority of patients.

Resveratrol is a compound found in the skin of red grapes. It is an effective antioxidant and protects against degeneration of neurons in ischemia. It is hypothesized that resveratrol is responsible for the “French paradox”, the observation that the French have a lower incidence of heart disease despite a high fat diet due to the consumption of red wine. It is an effective antioxidant and prevents neuron degeneration.^79^,^80^

## CONCLUSION

XFS is a systemic disorder with components of low-grade inflammation and oxidative damage leading to accumulation of XFM. The ocular morbidity associated with XFS far exceeds that of open-angle glaucoma, and a host of systemic diseases with considerable morbidity and mortality are associated with XFS. Further elucidation of the genetics and pathophysiology of XFS is crucial to our ability to treat this disease effectively. There is a role for non-IOP lowering treatment modalities in XFS as there is significant evidence of generalized ischemia and neurodegeneration. With this directed investigation, there is hope that XFS will be both a preventable and curable disease.
